# Selection of oleaginous yeasts for fatty acid production

**DOI:** 10.1186/s12896-016-0276-7

**Published:** 2016-05-27

**Authors:** Dennis Lamers, Nick van Biezen, Dirk Martens, Linda Peters, Eric van de Zilver, Nicole Jacobs-van Dreumel, René H. Wijffels, Christien Lokman

**Affiliations:** HAN BioCentre, University of Applied Sciences, P.O. Box 6960, , 6503 GL Nijmegen, The Netherlands; Bioprocess Engineering, Wageningen University and Research Centre, P.O. Box 8129, , 6700 EV Wageningen, The Netherlands; University of Nordland, Faculty of Biosciences and Aquaculture, N-8049 Bodø, Norway

**Keywords:** Oleaginous yeast, *Schwanniomyces occidentalis*, Lipid production, TAG

## Abstract

**Background:**

Oleaginous yeast species are an alternative for the production of lipids or triacylglycerides (TAGs). These yeasts are usually non-pathogenic and able to store TAGs ranging from 20 % to 70 % of their cell mass depending on culture conditions. TAGs originating from oleaginous yeasts can be used as the so-called second generation biofuels, which are based on non-food competing “waste carbon sources”.

**Results:**

In this study the selection of potentially new interesting oleaginous yeast strains is described. Important selection criteria were: a broad maximum temperature and pH range for growth (robustness of the strain), a broad spectrum of carbon sources that can be metabolized (preferably including C-5 sugars), a high total fatty acid content in combination with a low glycogen content and genetic accessibility.

**Conclusions:**

Based on these selection criteria, among 24 screened species, *Schwanniomyces occidentalis* (*Debaromyces occidentalis*) CBS2864 was selected as a promising strain for the production of high amounts of lipids.

**Electronic supplementary material:**

The online version of this article (doi:10.1186/s12896-016-0276-7) contains supplementary material, which is available to authorized users.

## Background

Mineral and vegetable oil is a crucial resource for the modern human civilization, but the worldwide amount is depleting rapidly and alternatives need to be explored. An interesting alternative to decrease the dependency of western societies on these fossil and vegetable sources might be the use of oleaginous micro-organisms as described by various authors [1–3]. Lipids isolated from oleaginous micro-organisms can be used as components in coatings, paints, personal care products, production of fine chemicals and biodiesel thereby decreasing the dependency on vegetable and mineral oil [4–7]. Fatty Acid Methyl Esters originating from the lipids of oleaginous micro-organisms (e.g. algae, yeast and fungi) show identical fuelling properties compared to conventional diesel and could be used in modern cars without major adaptations [8]. At this moment the majority of biodiesel is produced from lipids, which are also used in the food chain and thus compete with food for agricultural land [7]. Therefore, oleaginous micro-organisms growing on agricultural waste residues are an attractive class of micro-organisms for lipid production.

An interesting class of oleaginous micro-organisms are yeasts. Oleaginous yeasts are able to store large quantities of TAGs in the form of lipid bodies in the cells. Typical lipid contents range from 20 % to 76 % depending species and culture conditions. Oleaginous yeasts strains studied today are e.g. *Yarrowia lipolytica, Candida 107, Rhodotorula glutinis, Rhodosporidium toruloides, Cryptococcus curvatus, Trichosporon pullulan* and *Lipomyces lipofer.* Screening studies are still performed, leading to the identification of several new oleaginous yeast species [1, 9–11]. Lipid accumulation is triggered by a nutrient limitation combined with an excess of carbon. Mostly nitrogen limitation is used to trigger lipid accumulation, but also other nutrients as phosphorus and sulphur have been shown to induce lipid accumulation [12–15]. Oleaginous yeasts should preferably be able to grow to high cell densities combined with a high fatty acid content, have good growth characteristics at low pH and a broad temperature range (robust process conditions), which facilitate the process development for future industrial applications. Furthermore, the ability to grow on a broad spectrum of carbon sources make oleaginous yeasts economically interesting.

The aim of this study is to find new yeasts that meet the aforementioned criteria and are potentially suited for fatty acid production for industrial applications. To this extent 24 non-*Saccharomyces* yeast species were selected and tested for the above mentioned criteria. Some of these selected strains have been described as having an oleaginous character [10, 16–20].

After selection for growth rate, lipid accumulation capacity, ability to use different carbon sources, pH and temperature optimum, *Schwanniomyces occidentalis* was selected as the most promising strain.

## Results and discussion

### Selection of strains by TLC analysis

From a private collection 24 yeast strains were selected to investigate their possible oleaginous character, where for 4 of these strains 2 variants were included, resulting in a total of 28 yeasts tested (Table 1). Generally, it is considered that lipid accumulation is induced at a molar C/N ratio greater than 20 [20]. Previously, it was shown that lipid accumulation in *R. toruloides* is observed at a C/N ratio of 30 and increases with an C/N ratio up to 120 using glucose as carbon source [21]. When growing *Y. lipolytica* on glucose at a C/N ratio of 50 a lipid content of 36 % is reached [22]. In *T. cutaneum* a slight increase in lipid content was reached when increasing the C/N ratio from 60 to 180, followed by a sharp decrease when the C/N ratio was further increased to 200 [23]. Furthermore, for *C. freyschussi* similar lipid content was reached at a C/N ratios of 52 and 100 whilst an increase to C/N 200 had a negative effect on lipid content [24]. Not only the C/N ratio but also type of the carbon and nitrogen sources used can have an impact on lipid production [22, 25]. Therefore, in this study screening for novel oleaginous yeasts was performed using medium with a C/N ratio of 75, without optimizing growth conditions for each individual strain, using glucose as carbon and ammonium chloride as nitrogen source. The strains listed in Table 1 were cultivated in C/N 75 medium for three days. Cell mass was harvested and dry weight content and triacylglyceride content was determined after saponification.

**Table 1:** Strains used in this study

No.	Strain names	Culture collection
1	*Hansenula californica*	CBS 5760
2	*Candida glabrata*	CBS 2663
3	*Kluyveromyces phaffii*	CBS 4417
4	*Pichia angusta*	CBS 4732
5	*Torulopsis glabrata*	own collection/HBC14
6	*Pichia anomala*	CBS 5759
7	*Candida glabrata*	CBS 2192
8	*Candida lipolytica*	own collection/HBC08
9	*Torulopsis glabrata*	CBS 858
10	*Torulaspora delbrueckii*	own collection/HBC36
11	*Hansenula beijerinckii*	CBS 2564
12	*Candida tropicalis*	own collection/HBC07
13	*Yarrowia lipolytica*	CBS 6124
14	*Candida lusitaniae*	IFFI 01461
15	*Pichia silvicola*	CBS 1706
16	*Schwanniomyces occidentalis*	CBS 2864
17	*Sporobolomyces roseus*	CBS 2841
18	*Metschinikowia pulcherrima*	CBS 5534
19	*Candida bombicola*	ATCC 22214
20	*Candida intermedia*	CBS 572
21	*Candida tropicalis*	CBS 94
22	*Kloeckera africana*	CBS 277
23	*Pichia petersonii*	CBS 5556
24	*Lodderomyces elongisporus*	CBS 2605
25	*Kloeckera apiculata*	CBS 104
26	*Waltomyces lipofer*	CBS 5841
27	*Yarrowia lipolytica*	CBS 2073
28	*Crypotococcus curvatus*	CBS 570

**Fig. 1:**
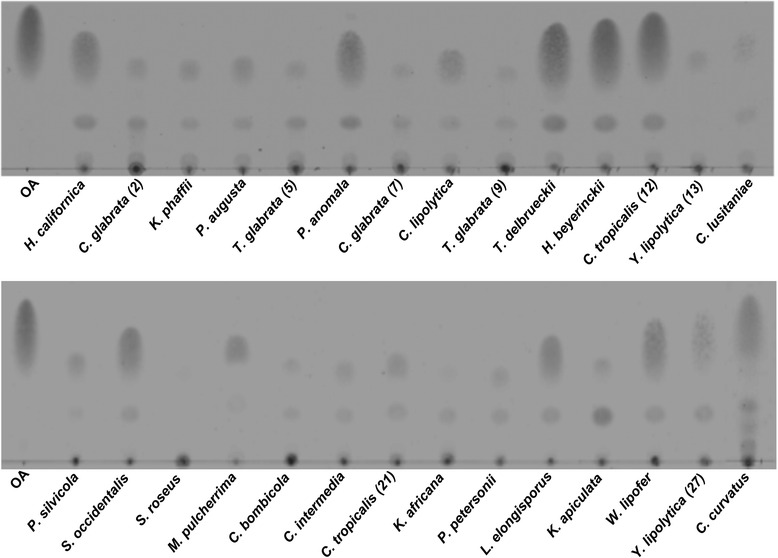
TLC chromatogram of fatty acids isolated after saponification of 27 screened strains; Strains were grown in shake flasks containing 30 ml medium of a C/N ratio of 75 at 30 °C. After 72 h total lipids were extracted followed by TLC analysis. The black spots indicate the fatty acid content per sample. Oleic acid (OA) is used as a positive control (C_18:1_). Each lane represents an equal amount of dry weight. When multiple variants of strains are used the number between brackets refers to the position of the strain variant in Table 1

In Fig. 1 the fatty acid content after saponification of the different strains is visualised by thin layer chromatography (TLC) using oleic acid as a positive control. Since equal amounts of dry cell mass were used, the intensity of the spot represents the triacylglyceride content per gram dry weight. From the TLC analysis, 10 strains could be identified as strains with a high triacylglyceride content, viz.; *H. californica, P. anomala, T. delbrueckii, H. beijerinckii, C. tropicalis (12), S. occidentalis, L. elongisporus, W. lipofer, Y. lipolytica (27)* and *C. curvatus.*

**Fig. 2:**
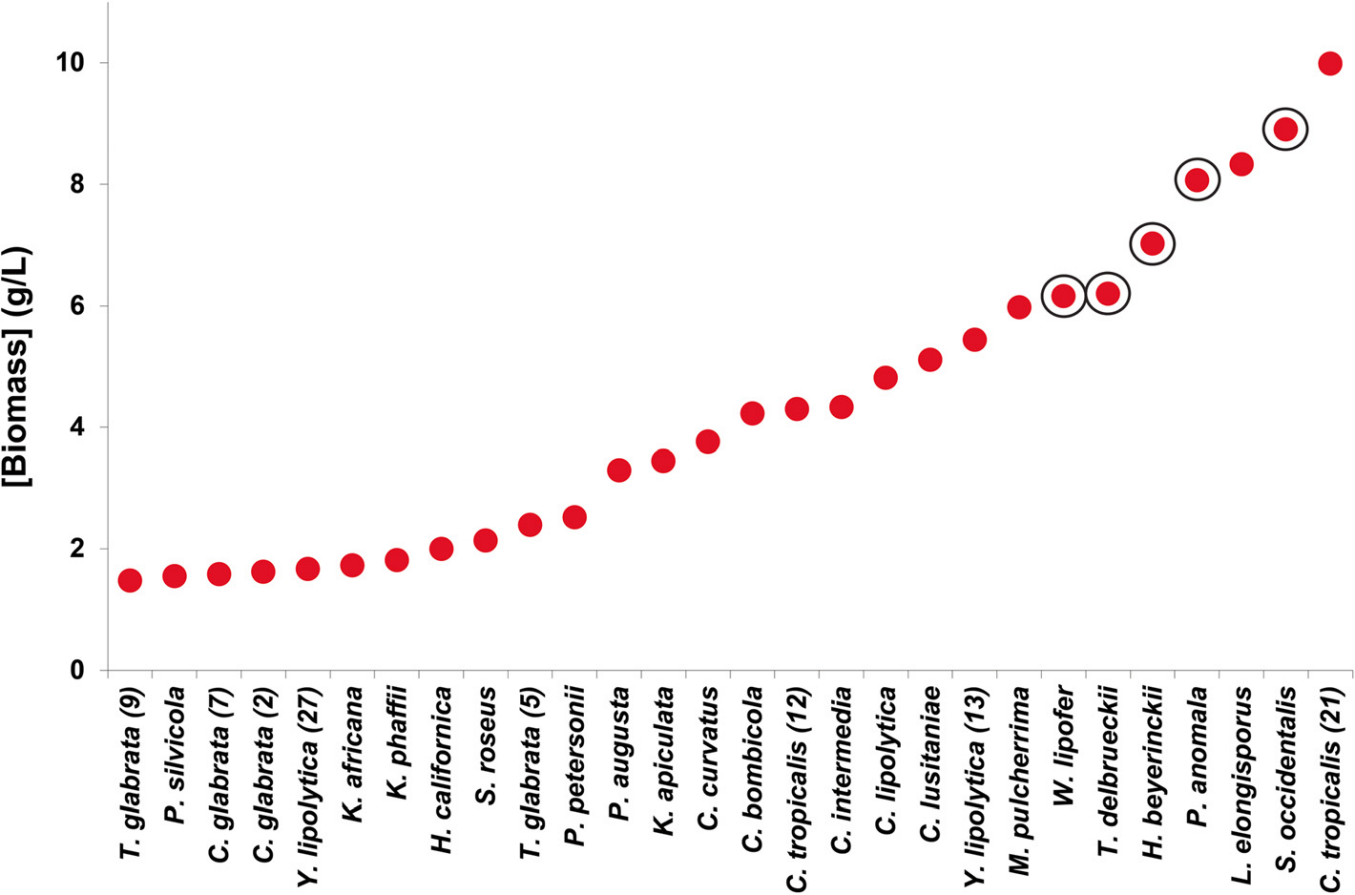
Final biomass mass concentrations after three days of growth; Strains were grown in shake flasks containing 30 ml medium of a C/N ratio of 75 at 30°. After 72 h of growth the biomass concentrations were determined. Strains are ordered in increasing amounts of biomass concentration. When multiple variants of strains are used the number between brackets refers to the position of the strain variant in Table 1. Five promising fatty acid producing strains are indicated with a circle

In Fig. 2 the final cell mass concentrations of the various strains are shown. Based on the results of Figs. 1 and 2, five strains were selected for further research, viz.; *P. anomala, T. delbrueckii, H. beijerinckii*, *S. occidentalis* and *W. lipofer*. Selection was based on a high content of triacylglycerides combined with a high cell mass concentration. The strain *L. elongisporus* met the criteria, but was not selected due to its suspected potential pathogenic character [26]. In addition based on literature *S. cerevisiae* was taken along as negative control.

### Growth of selected strains at various temperatures

Strains used in large scale production processes should preferably be robust. Robustness of a strain is defined as the possibility to withstand process disturbances (e.g. temperature and pH variations), without having a large influence on the productivity of the process. The effect of temperature (T) on cell growth was investigated for the selected yeast strains. A relatively broad T range at which the strains are still capable to grow without major changes in growth characteristics is desired in a large scale production process. In other words a shift in the temperature process control should have little effect on the final process. Furthermore, the ability to grow at higher temperatures is preferred to decrease the amount of cooling needed for cultivation [27].

**Fig. 3:**
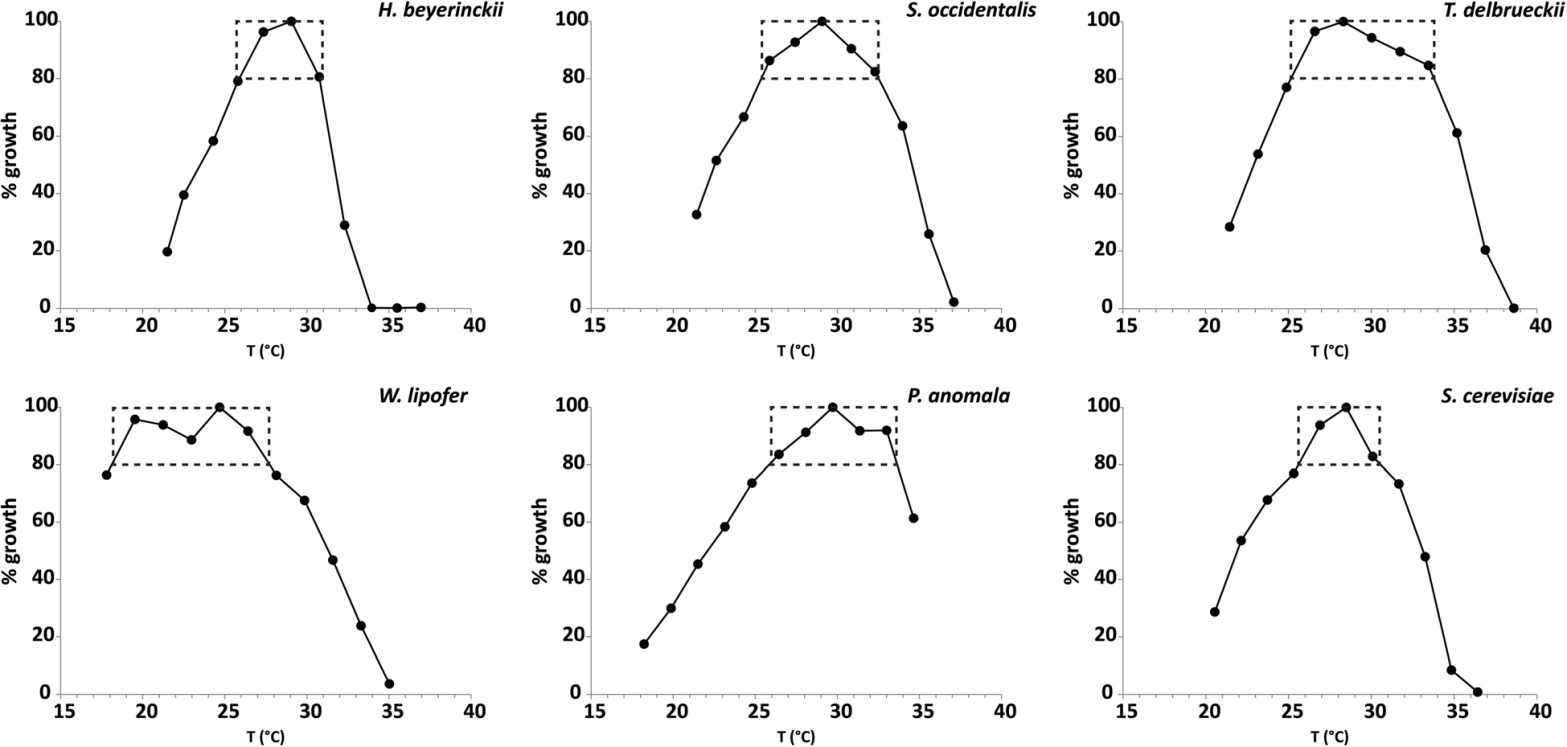
Growth profiles at different temperatures; Strains were grown in static 2 ml cultures using medium with a C/N ratio of 5 at varying temperatures. Growth was determined by measuring biomass concentrations. The highest biomass concentration was defined as 100 %. The dashed rectangle indicates the temperature range in which the specific growth rate is higher than 80 % of the maximum value. Depicted strains are *H. beijerinckii*, *S. occidentalis*, *W. lipofer*, *P. anomala*, *T. delbrueckii* and *S. cerevisiae*

Temperature profiles (Fig. 3) were obtained as described in the methods section. From these graphs two types of strains could be identified. Strains with a relatively narrow temperature range and strains having a broad temperature range in which growth is marginally influenced. Selecting the temperature area in which the growth rate is 80 % of the maximum value, it can be seen that strains *H. beijerinckii* and *S. cerevisiae* have a narrow optimum, with approximately a 5 °C bandwidth around the maximum growth. For all the other strains this bandwidth ranges from 7 °C to 9 °C, which is almost twice as broad (see dashed rectangles in Fig. 3). Based on the temperature profiles *S. occidentalis, P. anomala, W. lipofer* and *T. delbrueckii* are the most robust of the strains tested.

### Growth on different carbon sources

Production of bio-based materials should preferably be performed using renewable and low cost carbon sources (often harbouring C5-sugars e.g. xylose and arabinose). The results of the growth potential of all strains on various carbon sources is displayed in Table 2.

**Table 2:** Growth on agar plates containing different carbon sources

Carbon source	*S. occidentalis*	*W. lipofer*	*H. beijerinckii*	*P. anomala*	*T. delbrueckii*	*S. cerevisiae*
Glucose	+	+	+	+	+	+
Fructose	+	+	+	+	+	+
Galactose	+	+	+	+	+	+
Arabinose	+	+	-	-	-	-
Xylose	+	+	+	-	-	-
Glycerol	+	+	+	+	+	-
Sucrose	+	+	+	+	+	+
Lactose	+	-	-	-	-	-
Maltose	+	+	+	+	+	+
Cellobiose	+	+	+	+	-	-
Starch	+	-	-	-	-	-
Inulin	+	+	+	+	+	-
Xylan	-	-	-	-	-	-
Cellulose	-	-	-	-	-	-
No C source	-	-	-	-	-	-

From Table 2 it can be concluded that from the strains tested *S. occidentalis* can grow on all carbon sources used in this study except cellulose and hemi-cellulose, which requires the action of multiple enzymes: exo,1,4-β-D-glucanase; endo,1,4-β-D-glucanase; cellobiohydolase and β-D-glucosidase, which are not commonly found in yeast [28, 29]. However, also some typical fungal characteristics can be observed in this strain like growth on starch that can only be achieved in the presence of glucoamylase activity, which is normally present in filamentous fungi and absent in yeasts. These findings are in line with the presence of glucoamylase activity which was already confirmed in *S. occidentalis* [30]. Striking was also the growth on cellobiose requiring β-glucosidase activity, which is part of the cellulolytic enzyme activity of fungi but not of that of yeasts. The genome of *S. occidentalis* was sequenced and assembled and the presence of a β-glucosidase gene having an identity of 77 % with the β-glucosidase sequence of *Scheffersomyces stipitis* CBS 6054 (XP_001387646) was confirmed using tblastn. Growth on glycerol was tested, since it is an abundantly available side product obtained from biodiesel production. All strains except *S. cerevisiae* were able to grow on glycerol. The results of carbon source utilization by *S. occidentalis* correspond with a recent study in which different oleaginous yeast species were screened for carbon source utilization and inhibitory tolerance in order to select yeasts suitable for specific industrial applications [31]. In the aforementioned study of Sitepu et al., *S. occidentalis* was found to be resistant to inhibitors at concentrations that are common in lignocellulosic hydrolysates (e.g. 2 g/l HMF, 1 g/l furfural and 2,5 g/l acetic acid) thereby indicating its potential to utilize these carbon sources for lipid production.

Genetic engineering of oleaginous yeasts is frequently used to expand substrate utilization and further increase lipid content and productivity. The oleaginous yeast *Y. lipolytica* is unable to utilize starch and by the combined expression of alpha-amylase and glucoamylase growth on starch led to a fatty acid accumulation of 21 % which was further increased to 27 % after media optimizations [32]. Furthermore, Tai and Stephanopoulus report that co-expression of ACC1 and DGA1 increases fatty acid content from 8.77 % to 41.4 %% in *Y. lipolytica* [33]. A similar co-expression of ACC1 and DGA1 was performed in *R. toruloides* which increased lipid content from 31.3 % to 61.1 % [34]. Of the 5 strains tested only *S. occidentalis* is genetically accessible thereby indicating the potential to increase its fatty acid content, yield and carbon utilization [35, 36].

### Analysis of yeast cell mass

**Fig. 4:**
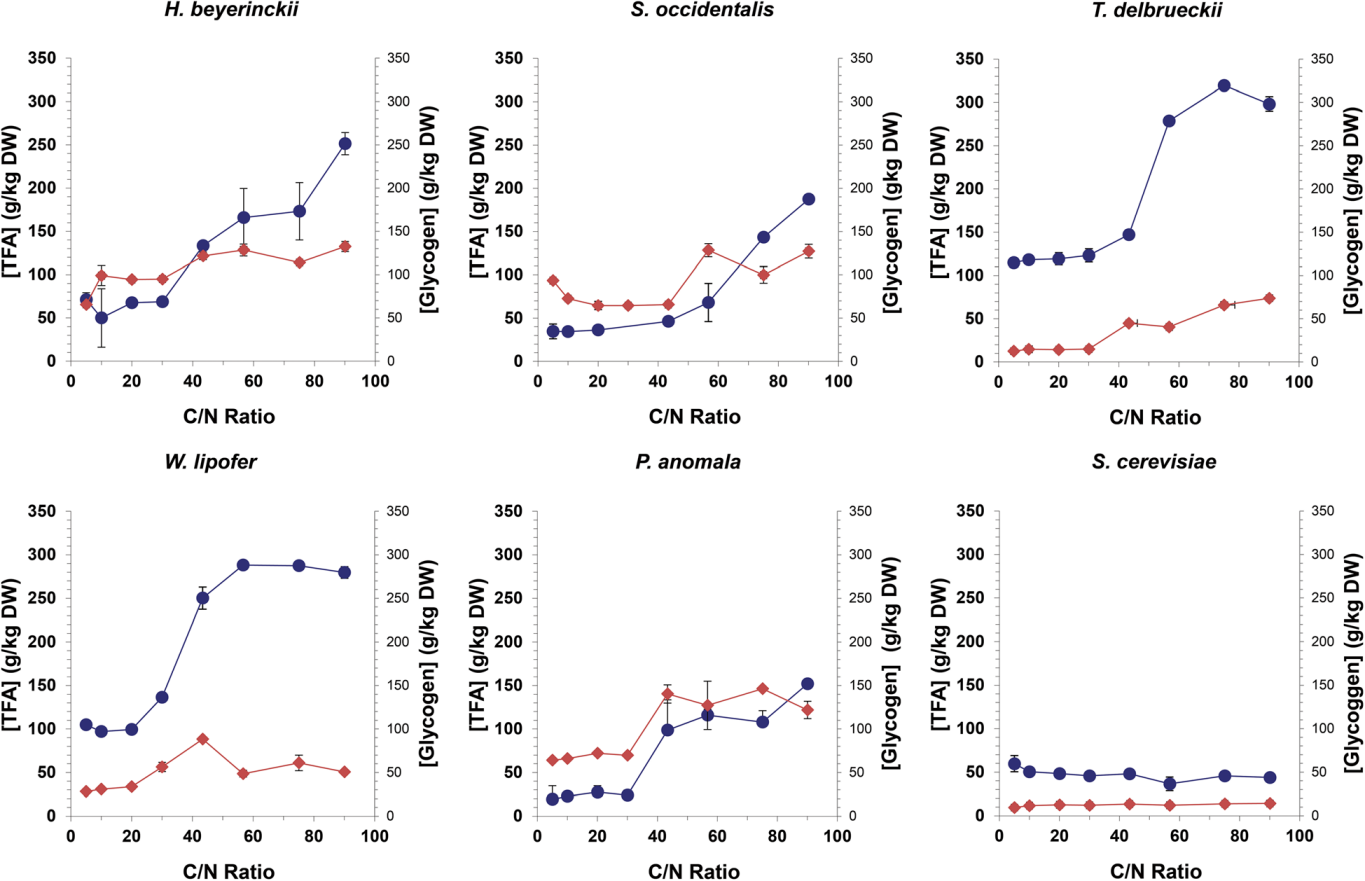
Total fatty acid and glycogen content at different C/N ratios; Strains were cultured at 30 °C and 300 rpm in 30 ml screw cap jars containing 10 ml medium of the desired C/N ratio. Total lipid an glycogen content was determined on 60 mg of freeze dried cells. Total lipid content (circle) and glycogen (diamond) shown for *H. beijerinckii*, *S. occidentalis*, *W. lipofer*, *P. anomala*, *T. delbrueckii*. and *S. cerevisiae* (negative control). Cultures were repeated in triplicate

The five selected strains and *S. cerevisiae* (negative control) were cultivated in media with different C/N ratios for three days. Cell mass was harvested and quantitatively analysed for triacylglycerides content using gas chromatography (see Additional file 1 for fatty acid composition of these strains). Results are shown as a function of the C/N ratio of the medium in Fig. 4. Fatty acid per dry weight content of all strains, except *S. cerevisae*, increased with an increasing C/N ratio. The maximum lipid accumulation was 319 g/kg reached by *T. delbrueckii* at a C/N ratio of 75 whereas the minimum lipid accumulation was 152 g/kg reached by *P. anomala* at a C/N ratio of 90. Typical fatty acid content reached by oleaginous yeasts is higher and ranges from 360 g/kg for *Y. lipolytica*, 580 g/kg for *C. curvatus* to 720 g/kg for *R. glutinis* [37]. The lower fatty acid content in this study could be attributed to the fact that cultivation conditions are not optimized. As previously demonstrated by Calvey et al. in *L. starkeyi* both the initial C/N ratio and the agitation rate can have an impact on lipid accumulation and by lowering the agitation rate from 300 rpm to 200 rpm lipid accumulation was increased from 28.43 % to 54.85 % [38]. Furthermore, in *L. starkeyi* medium optimization while utilizing a mixture of glucose and xylose increased lipid content from 38.8 % to 61.5 % indicating that optimal lipid production can be achieved by further optimizations [39]. However, it has to be noted that comparison of triacylglyceride content with other studies described in literature is difficult, since they are highly dependent on used culture conditions and C/N ratios.

To analyse the overall potential of triacylglyceride production the glycogen content per cell mass was also measured (Fig. 4). Cells undergoing nitrogen starvation are able to channel their carbon into several compounds such as lipids and glycogen [40]. Glycogen production under nitrogen starvation indicates that lipid concentrations could potentially be increased by channeling carbon from glycogen to lipid production via metabolic engineering or by changing culture condition [41]. All strains tested were able to accumulate glycogen at different C/N ratio’s. High glycogen contents were observed in *P. anomala, H. beyerinckii* and *S. occidentalis*, ranging from 125 g/kg to 146 g/kg. The *S. cerevisiae* strain contained low amounts of glycogen which is in line with the fact that in *S. cerevisiae* production of trehalose is favoured over glycogen under nitrogen depletion [42].

Taken together all of the aforementioned criteria e.g. lipid content, growth on carbon sources, temperature and genetic accessibility we selected *S. occidentalis* as the most versatile strain.

A fed-batch fermentation of *S. occidentalis* was performed starting with an initial C/N ratio of 75. Maximal lipid content reached in the fed-batch fermentation was 41.9 %, lipid productivity was 0,083 g/l.h while DCW produced was 19,01 g/l in 96 h (Table 3).

**Table 3:** Productivity of fed-batch fermentations of oleaginous yeasts grown on glucose

Strain	Substrate	Initial C:N ratio	Culture time (h)	DCW produced (g/l)	Lipids produced (g/l)	Lipid %	Lipid yield (g/g sugar)	Lipid productivity (g/l/h)	DCW productivity (g/l/h)	Reference
*S. occidentalis*	Glucose	75	96	19.00	8.00	41.90	0.120	0.083	0.198	This study
*L. starkeyi*	Glucose	72		18.28	10.03	54.85	0.170	0.060	0.112	[39]
*R. glutinis*	Glucose		50	110.00	70.04	64.00		0.950	1.408	[41]
*Y. lipolytica*	Glucose	50	48	43.38	15.93	36.73	0.200	0.904	0.332	[22]
*R. toruloides*	Glucose				52.20	58.60	0.200	0.360		[45]

In comparison the maximal lipid content in *S. occidentalis of 41.9 %* is higher than that of *Y. lipolytica 36.73 %* when grown in a fed-batch fermentation using glucose, however it has to be noted that the initial C/N ratio for *Y. lipolytica* was 50 [22]. Furthermore, the total amount of lipids produced by *L. starkeyi* on glucose at an initial C/N ratio of 72 is slightly higher than for *S.* occidentalis 8.00 g/l versus 10.03 g/l [38]. Both *R. glutinis* and *R. toruloides* surpass *S.occidentalis* both in lipid content (64.00 % and 58.60 % respectively) and in lipid productivity (0.950 g/l.h and 0.360 g/l.h respectively). Via medium optimization and genetic engineering both lipid content and productivity in *S. occidentalis* could be further increased [32, 39].

### Growth characteristics at low pH

Strains growing at relatively low pH values are more interesting, since infection problems can be reduced significantly in a large scale production process as can be seen in the dairy industry [43]. For this reason we investigated if *S. occidentalis* was able to grow by performing batch fermentations at a pH ranging from 2.5 to 7.5. and determining biomass after 24 h of growth. The results demonstrate that *S. occidentalis* was able to grow in the pH range from 3.5 to 6.5. Optimal growth was observed at a pH range of 4.5 to 6.5 with a biomass concentration of 10.5 g/l to 12.7 g/l, whereas biomass concentrations at pH 3.5 where 8.0 g/l.

## Conclusions

The aim of this study was to select strains with a high lipid production, broad temperature range for growth, the ability to use a wide variety of carbon sources and genetic accessibility. Selection based on these criteria resulted in the selection of *S. occidentalis* as the most promising strain for industrial applications due to its ability to grow at a broad temperature and pH range and the ability to utilize many different carbon sources. The fatty acid production was not optimized in this study and leaves room for further improvement by optimizing process conditions and via metabolic engineering.

## Methods

### Strains and media

Strains used in this study are described in Table 1. Strains were inoculated on YPD slants (1 % yeast extract, 2 peptone and 2 % glucose), grown at 30 °C and stored at 4 °C prior to use. A small amount of cells from the slants was resuspended in water, followed by centrifugation to remove any medium components. This cell suspension was used as an inoculum for growth experiments (typical seed rate was 0.01 %).

The composition of the C/N 75 medium (75 mol C/mol N) was: glucose.aq 33 g/l, NH_4_Cl 0.139 g/l, yeast extract (Gistex LS from DSM, @ 10 % N) 1.5 g/l, KH_2_PO_4_ 3.2 g/l, MgSO_4_.7H_2_O 1.0 g/l. Glucose was sterilised separately from the other medium ingredients (20 min, 121 °C). Filter sterilised biotin was added at a concentration of 0.02 mg/l. Adjustment of the C/N content of the medium was obtained by decreasing the NH_4_Cl content. With this yeast extract medium a C/N ratio of 90 mol C/mol N could be obtained, without varying the yeast extract content.

### Temperature gradient

Yeast strains were grown in static 2 ml cultures C/N 5 medium (addition of 9.7 g/l NH_4_Cl), using a temperature gradient block, which applies a gradient from 20 to 40 °C. After two days of cultivation the OD_600nm_ was measured. OD_600nm_ values were corrected for the amount of evaporation, which was determined by weighing the culture tubes prior and after cultivation. The growth was expressed as relative value against the maximum obtained value.

### Growth on various carbon sources

Agar plates were made from Yeast Nitrogen Base (YNB) medium (Roth art. no HP26.1) containing 2 % agar (Roth art. no 5210.1), which were supplemented with 2 % of the indicated carbon sources (see the results). Carbon sources were sterilised separately. Overnight cultures were diluted to an OD_600nm_ of 0.1 and 10 μl was spotted on the individual carbon source containing agar plates. Duplicate plates were incubated at 30 °C and checked daily for growth for 72 h. Growth in the spotted areas was analysed on plates with different carbon sources using plates lacking carbon source as a negative control.

### C/N ratio experiments

10 ml of the desired C/N medium was inoculated with a washed suspension of cells (typical seed rate was 0.01 %) and grown in 30 ml plastic flat bottomed screw cap jars (VWR art. no. 216–2694, diameter 3 cm and height 7 cm) with a cotton wool stopper in the cap. The jars were incubated on a rotary shaker (type Innova, New Brunswick Scientific) set at 300 rpm. After three days, the cells were harvested, washed and freeze dried for further analysis.

### Batch fermentations

Batch fermentations were performed in 7 l BioFlo115 fermenters using the C/N 5 medium. The pH was controlled with 2 M NaOH or 1 M H_3_PO_4_. During the fermentation process 4 ml samples were taken using an automatic sampler (Gilson art. no. F203B). Collected samples were cooled to 1 °C to quench the metabolism. Samples were used for dry weight determination and the presence of residual glucose was analysed on a Cobas Mira Plus using the Horiba ABX Pentra Glucose HK CP reagent (art. no. A11A01667).

### Fed batch fermenation

A single colony was used to inoculate 100 ml of C/N 75 medium. After 24 h growth at 30 °C and 250 rpm agitation the inoculum was transferred into a 1,25 L fermenter.

Fed-batch fermentation was performed in 1 l BioFlo 115 fermenter using the C/N 75 medium. After depletion of the initial glucose concentration a glucose feed of 1,1 g/h was established. The pH was controlled with 2 M NaOH or 1 M H_3_PO_4_. During the fermentation process 4 ml samples were taken using an automatic sampler (Gilson art. no. F203B). Collected samples were cooled to 1 °C to quench the metabolism. Samples were used for dry weight determination and the presence of residual glucose was analysed on a Cobas Mira Plus using the Horiba ABX Pentra Glucose HK CP reagent (art. no. A11A01667).

### Total lipids extraction and TLC analysis

500 μl of 50 % NaOH was added to a 2 ml cell suspension in capped glass tubes. Tubes were incubated overnight at 100 °C. After cooling down 1.1 ml of 37 % HCl was added to liquefy the soap. The mixture was extracted with 5 ml of ethyl acetate by vortexing, followed by centrifugation at 2000 g for 5 min. The top layer was transferred to another tube and the excess of ethyl acetate was evaporated by an air stream at 50 °C. After complete drying, 100 μl of ethyl acetate was added to dissolve the residue. To compare the strains, the residues were further diluted to obtain normalised concentrations according to their cell mass content. In total 30 mg of cells were used. Thin layer chromatography was used to separate and visualise the residues obtained from saponification. Diluted samples (10 μl) were loaded on TLC plates (MERCK art. no. code 1.05554.0001) and left at room temperature for drying. Plates were run in a pre-equilibrated TLC container with a mixture of hexane : ethyl acetate : acetic acid = 90 : 10 : 1 as the mobile phase. After 10 cm of front migration the plates were removed from the TLC container and air dried. To visualise the products the dried plates were sprayed with concentrated sulphuric acid : methanol = 1 : 1 and placed in an oven at 150 °C for approximately 30 min. Oleic acid was used as a control.

### Fatty acid determination with gas chromatography

The total fatty acid content was measured according to a modified version of the method described by Kang and Wang (2005). To 30 mg of freeze dried cells in a capped test tube with screw cap (VWR art. no. SCERE5100160011G1 and SCERKSSR15415BY100) 1 ml BF_3_/methanol reagent (Merck art. no. 8.01663.0500) and 1 ml heptane (Acros organics art. no. 120340025) was added. After overnight incubation at 70 °C 2 ml water was added and mixed. Subsequently, the tubes were centrifuged at 2000 g for 5 min, and the upper (heptane) layer was transferred to a GC vial and analysed on methylated fatty acids by using a Focus-GC (Interscience) equipped with FID. GC was equipped with a Stabilwax Column (Restek art. no. 10624) and uses hydrogen as carrier gas. The sum of the methylated fatty acids was quantified using methylheptadocanic acid as a standard.

### Glycogen analysis

Glycogen was analysed according to a modified method as described by Aklujkar et al. [44]. To 30 mg of dry weight cells 500 μl of 2 M NaOH was added followed by boiling for 1 h. To neutralize this mixture, 60 μl of 9 M H_2_SO_4_ and 600 μl 1 M NaAc/HAc buffer (pH 4,5) were added. To 400 μl of sample, 50 units of glucoamylase (Novozymes art. no. NS22035) were added and incubated at 50 °C for 1 h. The final glucose concentration of the mixture was measured with and without amyloglucosidase treatment on a Cobas Mira Plus using the Horiba ABX Pentra Glucose HK CP reagent (art. no. A11A01667). Maltose was used as a positive control to check the activity of the amyloglucosidase.

### Dry weight analysis

A culture sample of 10 ml was weighed on an analytical balance in a pre-weighed tube and centrifuged at 3500 g for 10 min. The cell pellet was washed with water, 20 % of the original volume, and centrifuged again (3500 g for 10 min.). The pellet was resuspended in 0.5 ml of water and frozen at −20 °C for 4 h. Cell pellets were dried by lyophilisation for 24 h on a Christ freeze dryer (type 2–4 LD) and dried pellets were weighed on an analytical balance. Biomass concentration is determined by dividing the weight of the dried biomass by the weight of the culture sample. After dry weight quantification, the freeze dried cells were used for total fatty acid and glycogen analysis.

## Abbreviations

C/N ratio, Molar ratio of carbon over nitrogen; FA, Fatty acid; FID, Flame ionization detector; GC, Gas chromatography; T, Temperature; TAGs, Triacylglycerides; TLC, Thin layer chromatography; YNB, Yeast Nitrogen Base; YPD, Yeast-Petone-Dextrose broth.

## Additional file

Additional file 1: Table S1. Fatty acid composition of strains cultured using medium with a C/N ratio of 75. Description: Fatty acid composition of tested strains depicted as % (w/w) of the total fatty acid levels. Experiments were performed in triplicate. (PDF 224 kb)
