# Supplementary material for: Selection of oleaginous yeasts for fatty acid production
Source: BMC Biotechnol. 2016 May 27;16:45. doi: 10.1186/s12896-016-0276-7 (PMC4884388; doi:10.1186/s12896-016-0276-7)
Supplement: Additional file 1: Table S1. — Fatty acid composition of strains cultured using medium with a C/N ratio of 75. Description: Fatty acid composition of tested strains depicted as % (w/w) of the total fatty acid levels. Experiments were performed in triplicate. (PDF 224 kb) [file 12896_2016_276_MOESM1_ESM.pdf]

Table S1: Fatty acid composition of strains cultured using medium with a C/N ratio of 75.

Fatty acid	Strain					
	H.beyerinckii	*S.occidentalis*	*T.delbrueckii*	*W.lipofer*	*P.anomala*	*S.cerevisiae*
C14	0.3 +/- 0.0	0.1 +/- 0.1	0.6 +/- 0.1	0.6 +/- 0.0	0.2 +/- 0.1	1.0 +/- 0.0
C16	17.1 +/- 0.1	14.5 +/- 0.2	9.3 +/- 1.2	15.4 +/- 1.1	19.1 +/- 2.5	18.2 +/- 0.3
C16:1	17.3 +/- 1.5	7.8 +/- 0.5	33.2 +/- 2.0	0.4 +/- 0.0	3.4 +/- 0.1	72.0 +/- 0.5
C18	0.5 +/- 0.1	1.4 +/- 0.0	1.9 +/- 0.1	4.4 +/- 0.8	3.8 +/- 0.6	6.8 +/- 0.1
C18:1	55.3 +/- 0.6	69.4 +/- 0.1	51.0 +/- 2.5	71.0 +/- 4.3	54.4 +/- 2.2	2.0 +/- 0.8
C18:2	7.8 +/- 1.7	4.8 +/- 0.4	3.7 +/- 0.8	6.2 +/- 2.0	16.7 +/- 0.2	0.0 +/- 0.0
C18:3	1.4 +/- 0.1	1.7 +/- 0.3	0.0 +/- 0.0	0.7 +/- 0.5	2.0 +/- 0.4	0.0 +/- 0.0
C20	0.0 +/ 0.0	0.0 +/- 0.0	0.1 +/- 0.0	0.2 +/- 0.0	0.2 +/- 0.2	0.0 +/- 0.0
C20:1	0.2 +/- 0.1	0.2 +/- 0.0	0.2 +/- 0.0	0.5 +/- 0.1	0.2 +/- 0.1	0.0 +/- 0.0
C20:4	0.0 +/- 0.0	0.0 +/- 0.0	0.0 +/- 0.0	0.0 +/- 0.0	0.0 +/- 0.1	0.0 +/- 0.0

Fatty acid composition of tested strains depicted as % (w/w) of the total fatty acid levels. Experiments were performed in triplicate.
